# Idelalisib-Induced Pneumonitis in Chronic Lymphocytic Leukemia

**DOI:** 10.7759/cureus.59541

**Published:** 2024-05-02

**Authors:** Sasikanth N Ravi, Irene W Choi, Eva K Ngapgue, Amanda G Stroiney, Clive J Miranda

**Affiliations:** 1 Pulmonology, Saint Peter’s University Hospital, New Brunswick, USA; 2 Internal Medicine, Abrazo Community Health Network, Phoenix, USA; 3 Internal Medicine, St. George's University School of Medicine, St. George's, GRD; 4 Internal Medicine, Saint Peter’s University Hospital, New Brunswick, USA; 5 Internal Medicine, University at Buffalo, Buffalo, USA

**Keywords:** progressive interstitial lung disease, drug-induced interstitial lung disease (di-ild), b-cell chronic lymphocytic leukemia (b-cll), mosaic, tree-in-bud, chronic lymphocytic leukemia (cll), hematology-oncology, phosphoinositide 3-kinase (pi3k), drug induced pneumonitis, idelalisib

## Abstract

Idelalisib, a phosphoinositide 3-kinase delta (PI3Kδ) inhibitor, effectively treats relapsed chronic lymphocytic leukemia (CLL). While this targeted approach offers a therapeutic edge, particularly in B-cell malignancies, it is associated with complications such as pneumonitis. This report details idelalisib-induced pneumonitis, highlighting the importance of early diagnosis and tailored treatment in achieving a favorable patient outcome.

## Introduction

Idelalisib, a PI3Kδ inhibitor, has transformed the management of relapsed and refractory chronic lymphocytic leukemia (CLL) by precisely targeting key pathways in B-cell malignancies [1,2]. This precision therapy enhances patient outcomes but requires diligent management of adverse effects, such as pneumonitis. Although pneumonitis is an infrequent side effect, affecting about 3% of patients on idelalisib, it presents a significant clinical challenge, as evidenced by various trials [2-4]. This rate is notably lower than that of fludarabine, another CLL therapy, where interstitial pneumonitis affects approximately 10% of patients [5]. This difference highlights the variability in pulmonary toxicity among CLL treatments, which can be explained by studying the interactions between mammalian/mechanistic target of rapamycin (mTOR) and PI3Kδ pathways, especially as the pneumonitis observed in idelalisib patients mirrors the effects seen with mTOR inhibitors [1].

Research into mTOR inhibitors reveals complex immune-mediated and dose-dependent mechanisms behind drug-induced pneumonitis (DIP). These mechanisms include activation of the innate immune system and a T-cell-mediated response under mTOR inhibition, which are not mitigated by corticosteroids, indicating a robust immune reaction to potential, yet unidentified, stimuli [6]. Additionally, the variability in DIP occurrences related to drug dosage underscores the need for precise dosing and careful monitoring when using these inhibitors. Intriguingly, retrospective studies in patients with metastatic renal cell carcinoma treated with mTOR inhibitors like everolimus or temsirolimus suggest a potential link between DIP development and improved clinical outcomes. The prolonged overall survival seen in patients with DIP suggests a complex relationship between drug-induced toxicity and therapeutic benefit [7]. This observation highlights the importance of further research into DIP's immune response dynamics and dosage optimization, which is particularly relevant for CLL treatments using idelalisib.

Essential strategies for managing idelalisib-related pneumonitis include assessing patients' pulmonary history before initiating treatment, especially those with pre-existing lung conditions. The National Cancer Institute's Common Terminology Criteria for Adverse Events (CTCAE) provides a framework to categorize the severity of pneumonitis, guiding clinicians in making informed decisions about dose adjustments or the need for corticosteroid intervention [8,9]. These guidelines are crucial for optimizing the safe use of idelalisib in CLL treatment, ensuring vigilant monitoring and timely therapeutic adjustments.

## Case presentation

A 71-year-old man with CLL treated with idelalisib since 2021, type 2 diabetes mellitus, and a history of cardiac arrest requiring a pacemaker following ibrutinib therapy presented to the emergency department (ED) in 2023 with progressive dyspnea on exertion ongoing for about two years. He had recently returned from the Philippines, where doctors treated him for pneumonia, a condition he had also encountered in 2021. Both presentations were without fevers, chills, cough, or phlegm production. The patient had no known personal or family history of pulmonary disease. The patient also denied any known exposure to birds, mold, farm animals, pets, farming supplies, or plastic manufacturing, agents commonly known to cause hypersensitivity pneumonitis.

**Figure 1:**
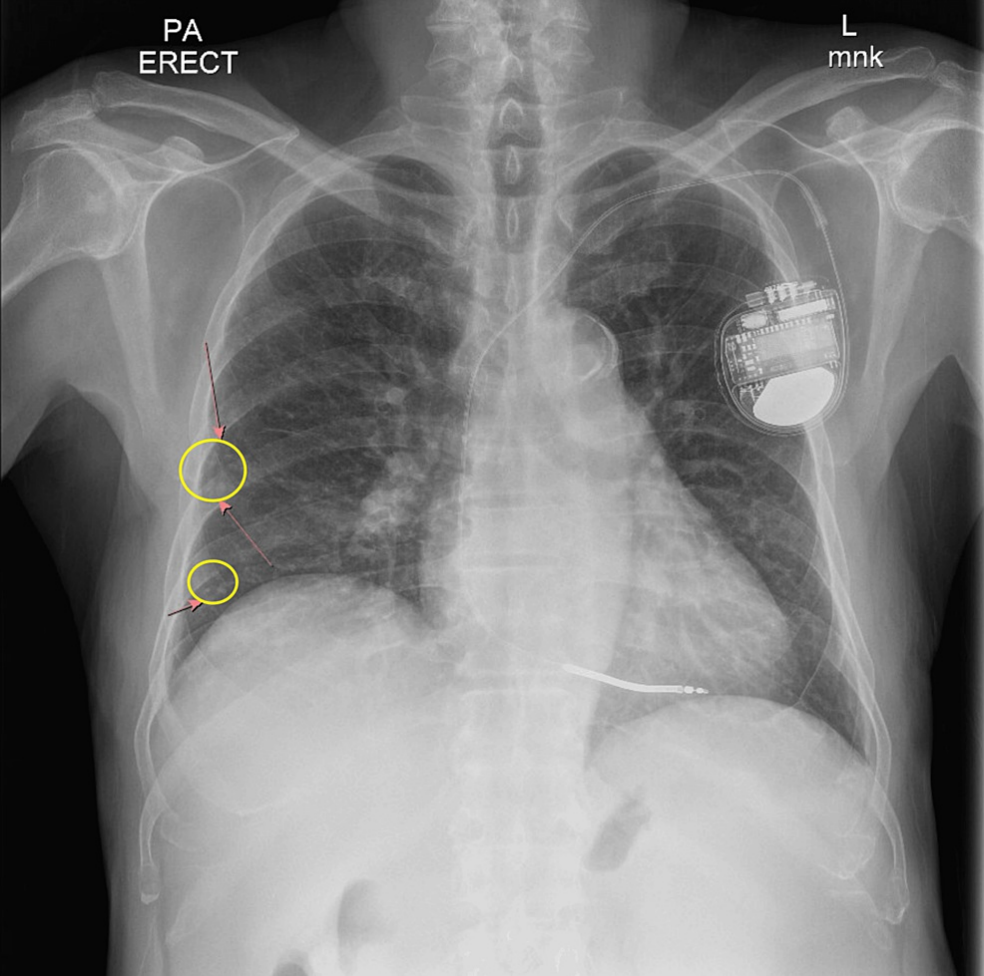
Chest X-ray illustrates tree-in-bud nodularity in the right middle (upper circle) and lower lobes (lower circle).

**Figure 2:**
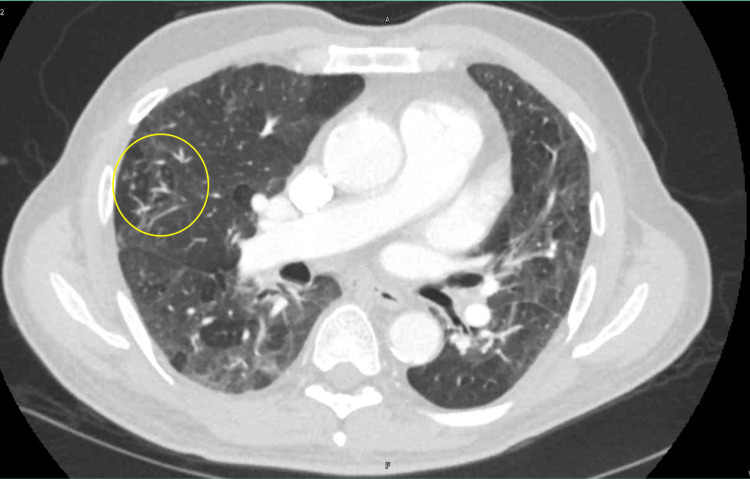
The CT thorax with contrast from 2023 highlights tree-in-bud nodularity in the right middle lobe (circle).

**Figure 3:**
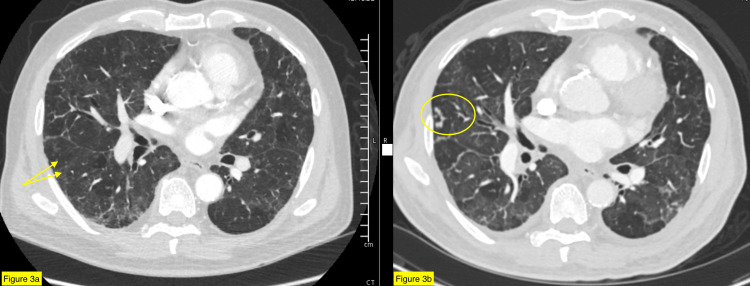
The CT thorax with contrast illustrates the progression of the diffuse mosaicism (arrows) and ground glass opacities with tree-in-bud nodular opacities (circle) from 2021 (Figure 3a) to 2023 (Figure 3b).

Laboratory tests revealed lymphocytic leukocytosis (WBC 16,700), anemia (hemoglobin 9.2 g/dL), thrombocytopenia (platelet count 87,000), and elevated ESR (64 mm/h). The chest X-ray showed tree-in-bud nodularity of the right middle lobe (Figure 1). Follow-up CT showed tree-in-bud nodularity within the right middle lobe and left upper lobe with an interval increase in the size of the nodular area and marked progression in the mosaic attenuation of the pulmonary parenchyma compared to imaging from 2021 (Figures 2-3). Cardiac evaluation, including troponin, pro-brain natriuretic peptide (BNP), and echocardiogram, were within normal limits. The infectious workup of procalcitonin, viral panel (including SARS-CoV-2, influenza, and respiratory syncytial virus), blood cultures, and QuantiFERON-TB were unremarkable.

After excluding other potential diagnoses and considering the timing of idelalisib initiation, the team diagnosed the patient with idelalisib-induced pneumonitis. This diagnosis stemmed from his clinical presentation, unrevealing laboratory findings, excluding other causes, and distinct radiological patterns. The patient's clinical improvement over three months following the discontinuation of idelalisib further confirmed the diagnosis. The medical team advised the patient to consult their oncologist for an alternative CLL treatment plan and to schedule a follow-up CT scan in three months to monitor radiological changes.

## Discussion

This case underscores the need to carefully manage idelalisib in CLL treatment, acknowledging PI3Kδ inhibitors' efficacy while recognizing the potential risk of inducing pneumonitis. The diagnosis, supported by distinct radiological changes post-idelalisib initiation and corroborated by clinical and laboratory findings, stresses the need for vigilant monitoring for pulmonary complications during CLL treatment. Despite idelalisib's efficacy, its association with pneumonitis, albeit rare at a 3% incidence rate, can affect lung function and quality of life [10]. Managed according to the CTCAE guidelines as grade 2 for non-life-limiting dyspnea, the patient's treatment involved discontinuation of idelalisib and subsequent outpatient monitoring [9]. The symptomatic improvement post-discontinuation validated the diagnosis and highlighted the importance of tailored management. The strategy of withholding glucocorticoids, in line with CTCAE recommendations for non-severe pneumonitis, endorses a measured approach, prioritizing a watchful waiting strategy for patients with stable or improving symptoms after ceasing the causative agent [11].

Moreover, the immunomodulatory effects of idelalisib, as demonstrated by its ability to reduce the expression of inhibitory checkpoint molecules in T cells and Tregs and to decrease T-cell-mediated cytotoxicity and cytokine secretion, underline the necessity of a cautious approach in its administration. These immunosuppressive actions contribute to an elevated risk of opportunistic infections, necessitating a comprehensive evaluation and monitoring strategy for patients under idelalisib treatment [1,12]. It is essential to recognize the dual nature of idelalisib's impact on immune function, balancing its therapeutic efficacy against the potential for immune-related adverse events and opportunistic infections. Thus, while managing idelalisib-induced pneumonitis, it is crucial to consider the broader spectrum of immunosuppressive effects, ensuring that clinicians take adequate measures to protect patients against the heightened risk of infections, particularly in the context of CLL's inherent immunocompromising condition. The CTCAE guidelines recommend this observant approach for symptoms not escalating to the severity of grades 3 or 4. At this level, glucocorticoid therapy becomes more pertinent, and managing potential infectious complications is crucial in the therapeutic strategy for patients receiving idelalisib [9,12].

## Conclusions

This case of idelalisib-induced pneumonitis in a CLL patient illustrates the necessity of balancing therapeutic benefits with potential adverse effects. It underlines the imperative for ongoing vigilance, timely intervention, and individualized management plans. The case also serves as a vital educational reference, emphasizing the need for heightened awareness among healthcare providers and patient education to optimize treatment efficacy and safety. Moreover, it invites further research into the suspected dose-related effects of mTOR inhibitors and their relation to the mechanisms of idelalisib-induced pneumonitis, aiming to refine CLL treatment protocols and enhance patient care outcomes.
